# Treating sepsis by targeting neutrophil extracellular traps: a systematic review and meta-analysis

**DOI:** 10.1186/s40635-026-00994-2

**Published:** 2026-11-04

**Authors:** Carley Fowler, Bahareh Razmand, Sebastian Togatorop, Miklós Lipcsey, Robert Frithiof, Stephanie Franzén

**Affiliations:** 1 Anesthesiology and Intensive Care, Department of Surgical Sciences, Uppsala University, Akademiska sjukhuset, Uppsala, Sweden; 2 Institute of Experimental Pathology (ExPat), Center for Molecular Biology of Inflammation (ZMBE), University of Münster, Münster, Germany; 3 Hedenstierna Laboratory, Department of Surgical Sciences, Uppsala University, Uppsala, Sweden; 4 Anesthesia and Intensive Care, Uppsala University Hospital, Uppsala, Sweden; 5 Department of Medicine, Division of Nephrology, Cardio-Renal Physiology and Medicine, University of Alabama at Birmingham, Birmingham, USA

## Abstract

**Background:**

Sepsis, a leading cause of death worldwide, is characterized by dysregulated host immune responses and multi-organ dysfunction. Neutrophil extracellular traps (NETs) are extracellular fibers from activated neutrophils that contribute to the immune response. However, excessive NETs formation during sepsis may exacerbate inflammation, thrombosis, and tissue injury, potentially affecting sepsis prognosis. We examined whether NETs-modulating interventions are beneficial in experimental sepsis models.

**Methods:**

Following PRISMA guidelines, this review analyzed studies from PubMed and Scopus that reported NETs-targeting therapies and included outcome measures. A meta-analysis of random-effects models using a restricted maximum likelihood estimation was performed with *a priori* moderators including NETs quantification tier, intervention timing relative to insult, and intervention class.

**Results:**

In 33 included studies, the therapies significantly reduced NETs levels, while three studies increased NETs following treatment. The *a priori* moderators, NETs tier and treatment timing, were not significant, whereas intervention class significantly modified treatment effects. The treated groups had a lower risk of death and reduced IL-6, IL-1β, TNF-ɑ levels when compared to the control group. The treatments reduced tissue injury with considerable heterogeneity (I^2^=68.4%) and should be cautiously interpreted due to small study numbers. NETs modulation was significantly associated with a decrease in cytokine concentration and tissue injury. The heterogeneity of protocols, diverse outcome measures, publication bias, and overall poor reporting quality of the studies warranted conservative effect estimates and cautious interpretation of findings.

**Conclusion:**

Our meta-analysis suggests NETs modulation is associated with reduced mortality risk, attenuated organ injury, and decreased inflammation, based primarily on LPS endotoxemia rodent models. Notably, experimental protocols and model limitations impede the translatability of these therapies for clinical care at this stage of research.

**Trial registration:**

In accordance with the guidelines, our systematic review protocol is registered with the International Prospective Register of Systematic Reviews (PROSPERO) registration number CRD420251124339 and was published with INPLASY on 20 August 2025

**Supplementary Information:**

The online version contains supplementary material available at https://doi.org/10.1186/s40635-026-00994-2.

## Introduction

Sepsis is a life-threatening condition resulting in organ dysfunction caused by a dysregulated host response to infection, as defined by the Third International Consensus Definitions for Sepsis and Septic Shock [1]. In 2017, sepsis accounted for nearly 50 million cases and 11 million deaths worldwide, making it a major global healthcare challenge [2]. Despite its impact, no single treatment has been proven to significantly reduce mortality. Current healthcare methods include controlling the source of infection, administering broad-spectrum antibiotics with fluids, and providing organ-supportive care [3].

Understanding the complex pathophysiology of sepsis and developing novel therapeutics is quite difficult. Experimental sepsis models predominantly use mouse and rat models, including but not limited to cecal ligation and puncture (CLP), injection of *E. coli* or lipopolysaccharide (LPS), cecal slurry, and pneumonia-derived sepsis [4, 5]. In large-animal models, researchers have used bacterial instillation and methicillin-resistant *S. aureus* into the lungs; intravenous administration of LPS and live bacteria; CLP; and inoculation of autologous feces into the abdominal cavity [5]. According to MQTiPSS guidelines, LPS is not an appropriate model of human sepsis. However, it continues to be the primary animal disease model, necessitating subgroups of LPS and infectious “true” sepsis models. Collectively, these animal models offer an essential framework for investigating the mechanisms of sepsis pathophysiology.

A highly active area of sepsis research focuses on modulating neutrophil extracellular traps (NETs). NETs are extracellular fibers from neutrophils that contain nuclear content such as histones and granular proteins [6]. Researchers have demonstrated that neutrophils form NETs through the NETosis pathway to trap and neutralize pathogens [7]. To identify and quantify NETs, scientists use specific biomarkers, including nucleosomes containing histone 3 (H3), myeloperoxidase (MPO), neutrophil elastase (NE), and cell-free DNA (cfDNA) [8–10]. Specifically, histone H3, released during cell death, contributes to neutrophil activation. Citrullination by peptidylarginine deiminase 4 (PAD4) generates citrullinated histone H3 (CitH3), a proposed early biomarker of sepsis [9]. Other markers, MPO and NE, are neutrophil granule enzymes involved in NETosis used to measure MPO–DNA and NE–DNA complexes as indicators of systemic inflammation [10]. cfDNA, released from NETs and damaged cells, acts as an indirect early sepsis marker [8]. Research shows that dysregulated NETs formation and clearance can worsen sepsis pathogenesis by amplifying inflammation, causing tissue injury, and promoting thrombosis [11]. However, the field still lacks clarity on how effectively NETs-targeted treatments improve patient outcomes. This knowledge gap continues to drive efforts to develop more comprehensive and effective therapies [12].

Therefore, we aimed to investigate whether the literature supports targeting NETs as a beneficial treatment strategy in experimental models. This systematic review and meta-analysis evaluated treatment effects on mortality, immune responses, and tissue injury in animal models of sepsis.

## Methods

We conducted a systematic review and meta-analysis of studies reporting the use of therapeutics targeting NETs in wild-type animal models of sepsis. Following the Preferred Reporting Items for Systematic Reviews and Meta-Analyses (PRISMA) guidelines, we created our search strategy and flow diagram. To evaluate bias and quality of the studies, we used the Systematic Review Centre for Laboratory Animal Experimentation (SYRCLE) method with the recommended support software, CAMARADES, and the Grading of Recommendations Assessment, Development, and Evaluation (GRADE) Framework [13, 14]. The International Prospective Register of Systematic Reviews (PROSPERO) was searched, and we did not find an ongoing or completed systematic review on NETs-targeted therapeutics for sepsis in animal models; therefore, the protocol was published with INPLASY (https://doi.org/10.37766/inplasy2025.9.0002) and the review was registered at PROSPERO 2025 (CRD420251124339).

### Data sources and search strategies

Literature search strategies were created using search terms related to sepsis research in animal models, with a focus on targeting NETs. We performed a literature review using two online databases (PubMed and Scopus) for records published between January 1, 2015, and May 31, 2025. The results of the literature search were exported to the reference management system Zotero for duplicate removal [15]. The search strategy and literature search were peer reviewed by C.F. and B.R.

### Inclusion and exclusion criteria

The inclusion criteria were as follows: (1) the therapy targeted NETs for treating sepsis; (2) the study involved a wild-type animal (all species/sex); (3) the study involved an animal model of sepsis or endotoxemia; and (4) the study was a randomized and nonrandomized animal study.

The exclusion criteria were as follows: (1) the study did not quantify NETs; (2) the study did not have a control group that was a disease model or a disease model with vehicle; (3) the study did not report endpoint outcomes; (4) the study did not report sample size; (5) *in vitro* study (6) not original research; (7) not written in English; and (8) non-accessible full article.

### Study selection

Using the search strategy to identify studies, the studies were exported to CAMARADES Software [16]. One review author (C.F.) independently performed a blinded screening process of the titles and abstracts of the retrieved articles for potential eligibility according to the inclusion criteria above. Any doubt about potential eligibility was resolved by discussion with B.R., S.T., and S.F. After completion, the methodological inclusion and exclusion decisions were summarized in a PRISMA flow diagram.

### Data extraction

After the initial screening, the selected articles were evaluated in full text by the three review authors (C.F., B.R., and S.T.) independently to decide whether they met the inclusion criteria. The extracted information was summarized in pre-specified data extraction tables. Any doubt of potential inclusion was resolved by discussion with S.F.

Due to the complexity of NETs quantification, NETs biomarkers were classified into tiers, with each study required to use one quantification method within that tier (Table 1). Tier 1 required co-localization staining of markers; tier 2 included single NETs components; tier 3 included indirect NETs biomarkers; and tier 4 included pathway biomarkers that do not directly measure NETs. If the study had more than one NETs marker, then the highest tier was chosen for further analysis. Due to the heterogeneity of experimental research, we first used the Cochrane method of vote counting, based on the direction of effect (Table 2). After the vote counting, endpoint outcome data were extracted from the control and treatment groups. In studies including multiple treatment groups, data were extracted from the treatment arm considered most clinically relevant and central to the study’s main hypothesis. For studies that expressed data graphically, WebPlotDigitizer version 5.2 software was used to extract data [17].

**Table 1:** NETs tier classification

Tier	Label	Description
1	Most specific	In-tissue co-localization of DNA + neutrophil granule proteins / citrullinated histones (e.g. Cit-H3 + MPO + DAPI IF).
2	Fairly specific	Single NETs-specific components or complexes without spatial info (e.g. Cit-H3 WB/IHC/ELISA; MPO–DNA or NE–DNA ELISA/SYTOX fluorescence).
3	Indirect/ non-specific	cfDNA/dsDNA alone; MPO or NE activity/protein alone; non-citrullinated histone H3 alone.
4	Mechanistic only	Pathway markers (e.g. PAD4, ERK, NOX2, ROS, etc.); not direct NETs measurement.

**Table 2:** Binary metric key

**Type of Therapy**	
Inhibitor of NETs	1
Degradation of NETs	2
Agonist of NETs	3
**NETs Quantification**	
Outcome	Binary Metric
Reduces NETs	1= Yes
	0=No
**Improvement Quantification**	
Outcome	Binary Metric
Reduce mortality	1= Yes
	0=No
Reduces proinflammatory cytokines	1= Yes
	0=No
Reduces damage in histology and pathology of tissue	1= Yes
	0=No

### Assessment of risk of bias (RoB) and GRADE

The included articles were assessed using SYRCLE’s RoB tool, a tailored version of the Cochrane RoB tool for bias in animal intervention studies. We followed ten questions derived from the SYRCLE RoBs and assigned a low, high, or unclear risk for each paper. The RoB questions include selection bias (sequence generation, baseline characteristics, allocation concealment); performance bias (random housing and blinding of assessors); detection bias (random outcome assessment and blinding of assessors); attrition bias (incomplete outcome data); reporting bias (selective outcome reporting); and other bias (Fig. 2). The certainty of evidence was assessed using a GRADE-adapted framework for preclinical studies according to the recommendations of Wei et al. [13] (Supplementary Figure 3)

### Statistical analysis

Meta-analyses were conducted depending on the dataset structure using the *metafor* package (version 4.8-0) in R (version 4.5.2)​. OpenAI ChatGPT (GPT-6; OpenAI, 2026) was used to assist with R code for the meta-analyses, which was independently reviewed and verified by the authors.​ Outcomes were analyzed separately by domain using a standard random-effects meta-analysis with restricted maximum-likelihood estimation (REML). For independence, one NETs effect size was included from each study based on the NETs marker with the highest methodological tier. For pooled cytokines, a multilevel model was used. For mortality outcomes, log risk ratios were derived from reconstructed 2×2 tables with a continuity correction of 0.5 applied when zero cells were present. Secondary outcomes were analyzed as standardized mean differences with a Hedges g correction and 95% confidence intervals (CIs). *A priori* moderators included the NETs tier, the timing of intervention parsed into continuous minutes, and the type of NETs modulation. Subgroup analyses and meta-regressions were conducted by including moderators as fixed effects, and the statistical significance of subgroup differences was evaluated using omnibus tests of moderators (QM statistic). A study-level meta-regression analysis assessed whether effect sizes for downstream outcomes (cytokine outcomes were grouped and averaged) were associated with NETs effect sizes. Heterogeneity was quantified using I [2] and Q values. Statistical significance was defined as p<0.05. Forest plots were generated for visual inspection of individual and pooled effect sizes. Publication bias was examined via funnel plots, Egger’s linear regression, and a trim-and-fill analysis [18]. Meta-analyses were performed conservatively due to review limitations.

## Results

### Study inclusion

**Fig. 1:**
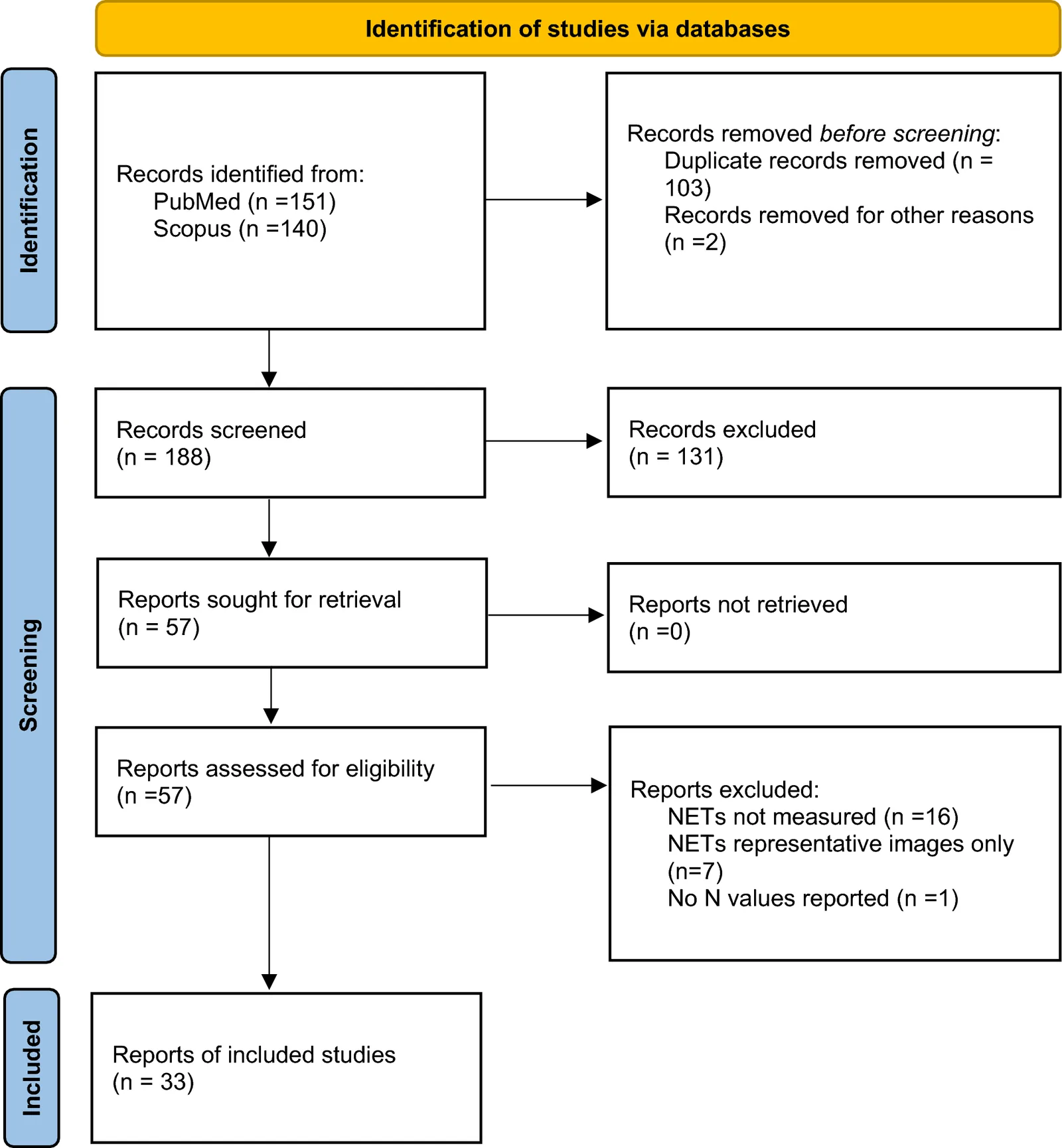
PRISMA flow diagram. The diagram illustrates the number of records identified, screened, excluded, and included in the final review, following PRISMA guidelines

The search strategy yielded 291 studies; 188 remained after duplicate removal, and redacted studies were excluded (Supplementary Figure 1). After screening the titles and abstracts, 57 articles met the inclusion criteria. After an in-depth review of the articles, exclusion criteria eliminated 24 reports, which generated 33 studies for the meta-analysis (Fig. 1, Supplementary Figure 2).

### Study characteristics

The search strategy included all animal models, but only studies using rats and mice met the inclusion criteria. For disease insult, 75% of the studies modeled sepsis by administering an intraperitoneal injection of LPS with doses ranging from 0.15 mg/kg to 35 mg/kg. Other studies induced sepsis via CLP, injection of *Pseudomonas aeruginosa*, or injection of cecal slurry. Regarding intervention characteristics, most studies used NET or NETosis inhibitors, fewer used NET degraders, and only three evaluated NET agonists. Due to the nature of pre‑clinical research, 45% of the studies administered the intervention before inducing sepsis and used a wide range of doses, spanning from 1 mg/kg to 1800 mg/kg. To quantify NETs, most studies relied on tier 2 methods. Investigators used a variety of outcome measures, including survival, cytokine levels, and histological evaluation. They typically assessed continuous outcomes early; 78% of studies collected data between 12 and 24 hours. In contrast, survival outcomes were assessed over longer periods, with 65% of studies analyzing survival beyond 96 hours (Table 3, Table 4, Table 5).

**Table 3:** Study design

Study number	Study (author, year)	Species, strain, gender	N per group	Sepsis model
1	Cai et al. (2024)	Mice C57BL/6J NR	6-8	LPS
2	Liu et al. (2023)	Mice C57BL/6 Male	3	LPS
3	Ishikawa et al. (2017)	Mice C57BL/6J Male	3-6	LPS
4	Zhang et al. (2024)	Mice C57BL/6 Male	4	CLP
5	Zhang et al. (2022)	Mice C57BL/6 Male	5-8	LPS
6	Lu et al. (2025)	Mice SPF BALB/c Male	3	CLP
7	Pan et al. (2017)	Mice C57BL/6J Male	3	LPS
8	Deng et al. (2019)	Mice C57BL/6 Male	3	LPS
9	Kwak et al. (2024)	Mice BALB/c NR	8	LPS
10	Zheng et al. (2023)	Mice C57BL/6J Male	6	LPS
11	Chen et al. (2023)	Mice C57BL/6 Male	6	LPS
12	Yao et al. (2022)	Mice C57BL/6J Female	4-11	LPS
13	Wu et al. (2020)	Mice C57BL/6J Male	3-8	LPS
14	Liang et al. (2018)	Mice C57BL/6J NR	3-8	LPS
15	Ni et al. (2021)	Mice C57BL/6J NR	8	CLP
16	Pei et al. (2022)	Mice mixed 129/SvJ and C57BL/6 NR	5-10	LPS
17	Jeong et al. (2022)	Mice C57BL/6 NR	3	LPS
18	Fang et al. (2025)	Mice C57BL/6J Male	4	LPS
19	Yan et al. (2024)	Mice C57BL/6J Male	6	LPS
20	Fei et al. (2024)	Mice C57BL/6 Male	6	LPS
21	Colón et al. (2019)	Mice C57BL/6 Male	4-5	LPS
22	Xiang et al. (2024)	Rats Sprague-Dawley Male	4-6	LPS
23	Gao et al. (2015)	Rats Sprague-Dawley Male	4-6	LPS
24	Czaikoski et al. (2016)	Mice C57BL/6 NR	5	CLP
25	Campbell et al. (2021)	Mice C57BL/6 NR	16	Cecal slurry injection
26	Wang et al. (2021)	Mice C57BL/6 Male	6	LPS
27	Song et al. (2022)	Mice C57BL/6J Male	4-6	LPS
28	Dong et al. (2025)	Mice C57BL/6J Male	3-5	*Pseudomonas aeruginosa*
29	Liang et al. (2025)	Mice C57BL/6 Male/Female	3-5	LPS
30	Kim et al. (2024)	Mice C57BL/6 Male	4-8	LPS
31	Araújo et al. (2016)	Mice Swiss Male	6-10	CLP
32	Hosoda et al. (2017)	Mice BALB/c Male	16-17	CLP
33	Ganatra et al. (2017)	Mice C57BL/6 Male/Female	8	Cecal slurry injection

**Table 4:** Therapy characteristics

Study number	Therapy	Type of therapy	Therapy dose	Timing relative to insult
1	Alpha-1 antitrypsin	Inhibitor	100 mg/kg	24h and 1h before LPS
2	Alpha-linolenic acid	Inhibitor	1800 mg/kg	1h before LPS
3	Antithrombin III	Inhibitor	250 IU/kg	3h after LPS
4	Atorvastatin + Imipenem	Inhibitor	1 mg/kg + 25 mg/kg	6h and 18h after CLP
5	Auricular Vagus Nerve Stimulation	Inhibitor	15 mins	30 mins before LPS
6	GSK 484-H	Inhibitor	4 mg/kg	daily for 7 days before LPS
7	YW3-56	Inhibitor	5mg/kg	in parallel with LPS
8	CitH3 mAb (4 Cit)	Inhibitor	20 mg/kg	right after LPS
9	AR-Dnase1	Degrader	3.75 mg/kg	2h after LPS
10	Anti-CD64 + BMSCs-EXO-NC	Inhibitor	100mg	24h before LPS
11	Hesperetin	Inhibitor	50 mg/kg	daily 7 days before LPS
12	YW3-56	Inhibitor	10 mg/kg	30 mins before LPS
13	AFM32a	Inhibitor	20mg/kg	1h after LPS
14	YW3-56	Inhibitor	5 mg/kg	30 min after LPS
15	Sivelestat + ITEM2	Inhibitor	100 mg/kg + 0.5 mg/kg	3-day intervals before CLP challenge at 1 h
16	Lysophosphatidic Acid Receptor 3 Inhibitor	Inhibitor	15 mg/kg	1h after LPS
17	Lysophosphatidylcholine	Inhibitor	10 mg/kg	3h after LPS with 4 injections at 12 h intervals for 48 h
18	Mac-1 inhibitory antibody	Inhibitor	100mg	30 mins before LPS
19	Medical ozone	Degrader	15 mg/mL	2h before LPS
20	Sivelestat	Inhibitor	200mg/kg	1h after LPS
21	DNase I	Degrader	10 mg/kg	10 min and 4 h after LPS then every 12 h thereafter
22	DNase I	Degrader	10 mg/kg	1h after LPS
23	DNase I	Degrader	10 mg/kg	30 mins after LPS
24	DNase I + ertapenem antibiotic	Degrader	10 mg/kg + 30 mg/kg	DNase1:1h and every 8h after CLP + antibiotic: 1h and every 12h after CLP
25	A1AT cleavage fragment	Inhibitor	10 mg/kg	1h before and 4h after
26	Ethyl pyruvate	Degrader	100mg/kg	1h before LPS
27	PTP1B inhibitors	Inhibitor	10 mg/kg	24h after LPS
28	hCitH3-mAb + paquinimod	Inhibitor	10mg/kg + 5mg/kg	30 min after PA
29	Rosa roxburghii Tratt	Inhibitor	500 mg/kg	7 days before LPS
30	SH003	Inhibitor	400 mg/kg	3 times a week for 2 weeks before LPS
31	Rosiglitazone	Agonist	0.5 mg/kg	15 min after CLP
32	LL-37	Agonist	2 μg/mouse	Before CLP
33	Zinc gluconate solution	Agonist	10 mg/kg	Daily for 3 days before ​

**Table 5:** NETs assessment and outcomes

Study number	Tier of NETs quantification	Continuous outcomes	Timing of outcome assessment
1	3	1.TNF-α​ 2.IL-6 3.IL-1β 4, Survival	Survival: 20h Continuous: 8h
2	2	1.IL-1β 2. Histology	Continuous:24h
3	1	1.TNF-α​ 2.IL-6 ​ 3.. Histology 4. Survival	Survival: 60h Continuous: 18h
4	1	1.TNF-α​ 2.IL-6 3.IL-1β 4. Histology 5. Survival	Survival: 168h Continuous: 24h
5	1	1.TNF-α​ 2. Histology	Continuous: 12h
6	2	1.IL-6 2.IL-1β 3. Histology	Continuous: 12h
7	2	1.IL-6 2.IL-1β	Continuous: 12h
8	2	1.TNF-α​ 2.IL-1β 3. Histology 4. Survival	Survival: 240h Continuous: 12h
9	2	1. Survival	Survival: 80h Continuous: 6h
10	2	1.TNF-α​ 2.IL-6 3.IL-1β 4. Histology	Continuous: 24h
11	1	1.TNF-α​ 2.IL-6 3. Histology	Continuous: 24h
12	2	1.TNF-α​ 2.IL-6 3.IL-1β 4. Survival	Survival: 240h Continuous: 24h
13	2	1.TNF-α​ 2.IL-1β 3. Histology 4. Survival	Survival: 240h Continuous: 24h
14	2	1.Histology 2. Survival	Survival: 240h Continuous: 12h
15	2	1.IL-1β 2. Histology 3. Survival	Survival: 168h Continuous: 144h
16	2	1.IL-6 2. Histology 3. Survival	Survival: 64h Continuous: 64h
17	1	1. Histology	Continuous: 48h
18	1	1.TNF-α​ 2.IL-6 3.IL-1β 4. Histology 5. Survival	Survival: 96h Continuous: 8h
19	3	1.IL-1β 2. Survival	Survival: 72h Continuous: 12h
20	3	1.Histology 2. Survival	Survival: 72h Continuous: 6h
21	2	1.TNF-α​ 2. Survival	Survival: 120h Continuous: 6h
22	1	1.TNF-α​ 2.IL-6 3.IL-1β 4. Histology	Continuous: 24h
23	2	1.TNF-α​ 2. Histology	Continuous: 24h
24	3	1.TNF-α​ 2. Survival	Survival: 168h Continuous: 12h
25	2	1. Survival	Survival: 96h
26	1	1.TNF-α​ 2.IL-6 3. Histology	Continuous: 24h
27	1	1.Histology 2. Survival	Survival: 120h Continuous: 24h
28	2	1.TNF-α​ 2.IL-6 3.IL-1β 4. Histology 5. Survival	Survival: 250h Continuous: 18h
29	2	1.TNF-α​ 2.IL-6 3.IL-1β 4. Histology 5. Survival	Survival: 168h Continuous: 24h
30	2	1.TNF-α​ 2.IL-6 3.IL-1β ​ 4.. Histology. 5.. Survival	Survival: 168h Continuous: 24h
31	3	1.TNF-α​ 2.IL-6 3.Survival	Survival: 168h Continuous: 24h
32	2	1. TNF-​α 2.IL-1β 3. Survival	Survival: 24h Continuous: 20h
33	1	1. IL-6 2.IL-1β 3.Survival	Survival: 72h Continuous: 6h

### Risk of bias and GRADE assessment

**Fig. 2:**
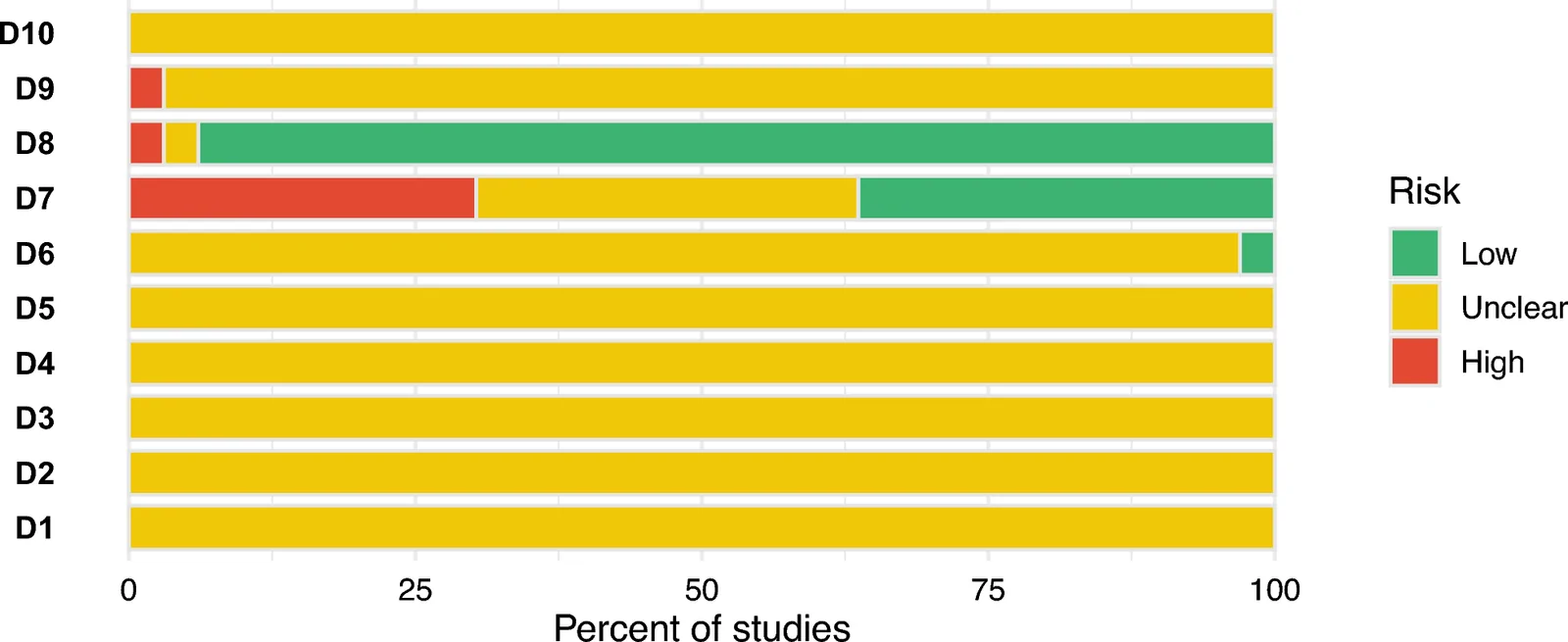
Risk of bias summary. The figure shows the percentage of studies judged to be at low (green), unclear (yellow), or high (red) risk of bias for each domain of the SYRCLE tool

Although some studies reported randomization of animals, none reported use of random generators; thus, selection bias (D1-D3) was "unclear" for all studies. For performance bias (D4, D5), the methods did not describe random housing or blinding of caregivers/investigators. For detection bias (D6), 1 study reported random selection of image fields. For D7, 36% of studies were at high risk of outcome bias because subjective outcomes were assessed without blinding. 33% of studies were graded unclear for objective outcomes that lack an explicit blinding statement, while 30% of studies for histological scoring were classified as low risk. For incomplete outcome data, one study was declared unclear and one high risk; otherwise, the studies were at low risk (Fig. 2). Reporting bias and other biases were difficult to assess due to the absence of protocol registration and limited methodological details. For the GRADE assessment, all outcomes were considered to have low overall certainty of evidence, except mortality, which was rated as moderate certainty because it did not show inconsistency (Sup. Fig. 3).

**Fig. 3:**
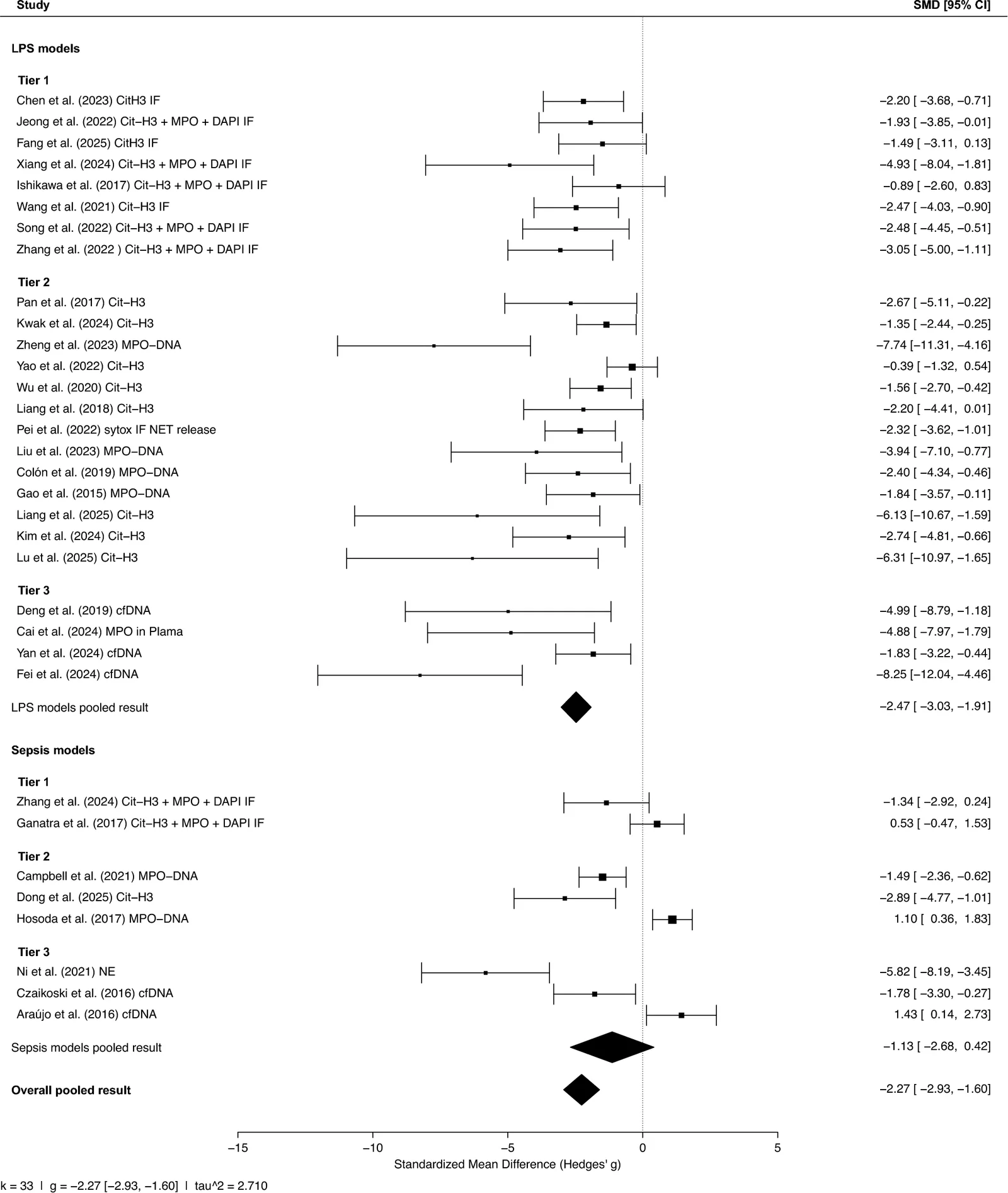
Forest plot of the NETs biomarkers stratified by tiers. The plot shows effect sizes, with negative g values indicating a decrease in NETs marker expression, along with their 95% confidence intervals. The black square represents the SEM (Hedge g) for each study, and its size is proportional to the study’s statistical weight. The black diamond presents the pooled random-effect size for this model

### NETs

The researchers quantified NETs using various techniques, including CitH3+MPO+DAPI immunofluorescent colocalization, MPO–DNA or NE–DNA complexes, CitH3, MPO, and cfDNA. For clarity, we grouped the studies into the defined tiers based on the specificity of NETs quantification. In total, 30% of the studies were tier 1, 55% were tier 2, and 15% were tier 3. Overall, the therapies in the LPS and sepsis groups showed a consistent and significant reduction in NETs biomarker levels, although 3 studies reported increased NETs levels (Fig. 3; g=-2.27; 95% CI:[-2.93,-1.60]). Consistent with MQTiPSS guidelines, an analysis with only the sepsis models (all non-LPS models) showed a non-significant reduction in NETs levels, with 3 studies reporting increased NETs levels (k=8; g=-1.13; 95% CI:[-2.68,0.42], p=0.151). There is significant heterogeneity among the studies (Q(32)=159.8, p<0.001) with variability (I^2^=81.2%, τ^2^=2.71). Subgroup analysis examining tier classification and timing relative to insult as moderators did not demonstrate a significant difference (QM(2)=1.35, p=0.508; QM(2)=1.19, p=0.550). In contrast, NETs-modulation type significantly moderated treatment effects (QM(2)=27.99, p<0.001) and appears to be the main driver of heterogeneity. In sensitivity analyses restricted to therapeutic treatments, the pooled reduction in NETs effect size remained large and statistically significant (g=-1.85, 95% CI:[-2.53, -1.17], p <.0001).

### Mortality

**Fig. 4:**
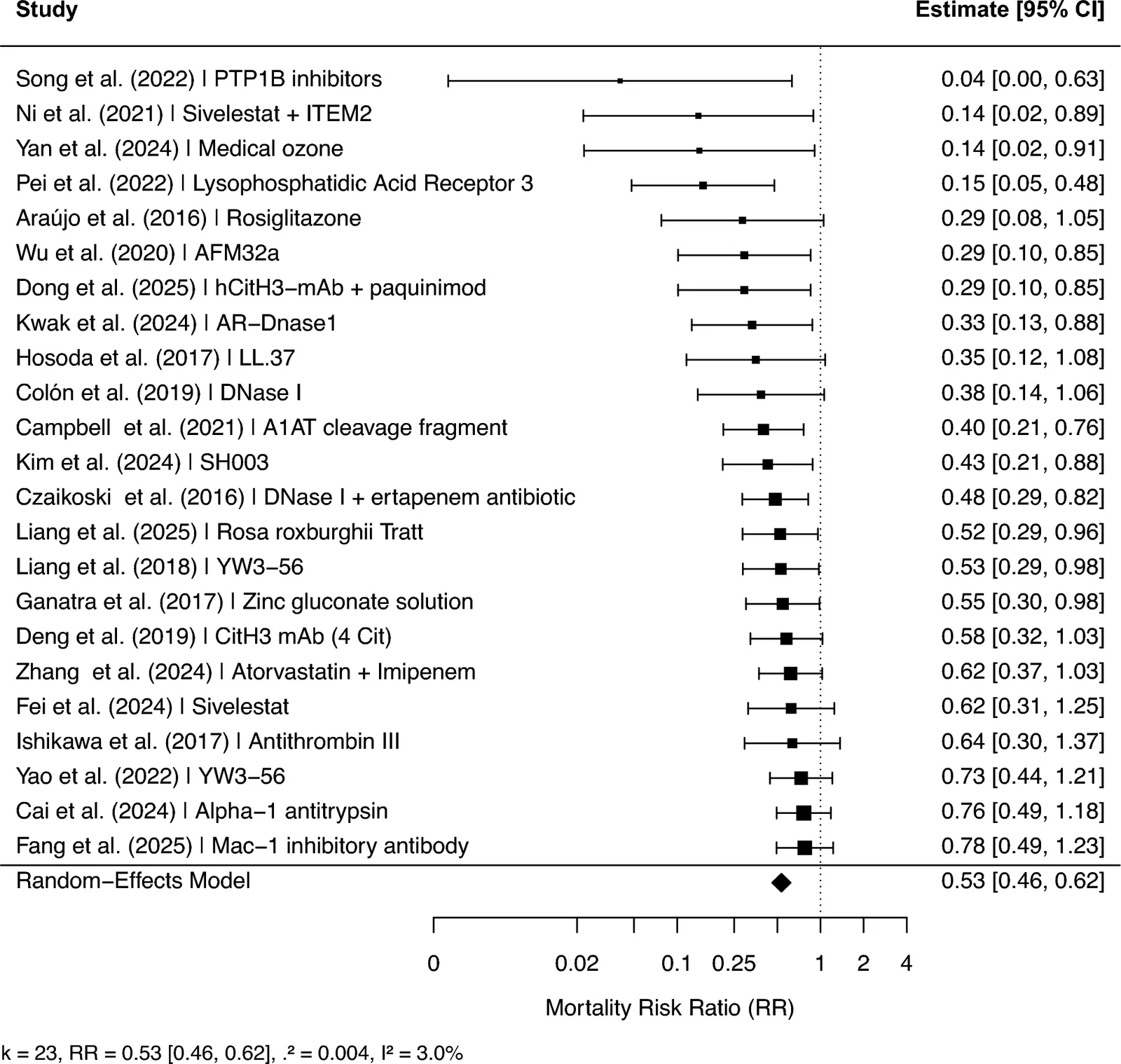
Forest plot of mortality risk ratio. Squares represent individual study estimates, and horizontal lines denote 95% confidence intervals. The diamond represents the pooled random effects estimate. An RR<1 indicates reduced mortality in treated animals compared with controls, while an RR>1 indicates increased mortality. The vertical reference line corresponds to RR = 1

Studies evaluating survival outcomes showed that therapies for NETs modulation were consistently associated with reduced mortality. In this review, 23 studies reported survival outcomes, and the pooled meta-analysis demonstrates that NETs-modulating therapies were associated with decreased risk of death (Fig. 4, pooled RR 0.53; 95% CI:[0.46,0.62], Z=-8.29, p<0.001). Between-study heterogeneity was low and not significant (I^2^=3%; τ^2^=0.004; Q(22)=25.9, p=0.255). In the true sepsis model subgroup, there was a significant reduced mortality risk (RR 0.47; 95% CI:[0.36, 0.60]; Z=-5.80, p<0.001). Further subgroup analyses demonstrated reduced mortality across all disease models, therapy classes, and intervention timings with no significant subgroup effects (Table 6). A meta-regression found no significant association between mortality assessment time and mortality RR (β = -0.089, 95% CI:[-0.28, 0.098], p = 0.350). Similar non-significant findings in the sensitivity analyses using raw follow-up duration and categorical follow-up groups (data not shown).

**Table 6:** Mortality subgroup analyses

Variable	Subgroup	Number of studies	RR (95% CI, p-value)	p-value of subgroup effect
Disease Model	LPS	15	0.57 (0.47–0.68) p<0.001	0.20
	Sepsis	8	0.47 (0.36–0.60) p<0.001	
Type of modulation	Inhibitor	16	0.57 (0.48–0.67) p<0.001	0.28
	Degrader	4	0.41 (0.27–0.62) p<0.001	
	Agonist	3	0.46 (0.28-0.75) p=0.0017	
Intervention timing	Prophylactic	10	0.59 (0.48-0.73) p<0.001	0.14
	Therapeutic	13	0.48 (0.39-0.59) p<0.001	

### Pro-inflammatory cytokines

**Fig. 5:**
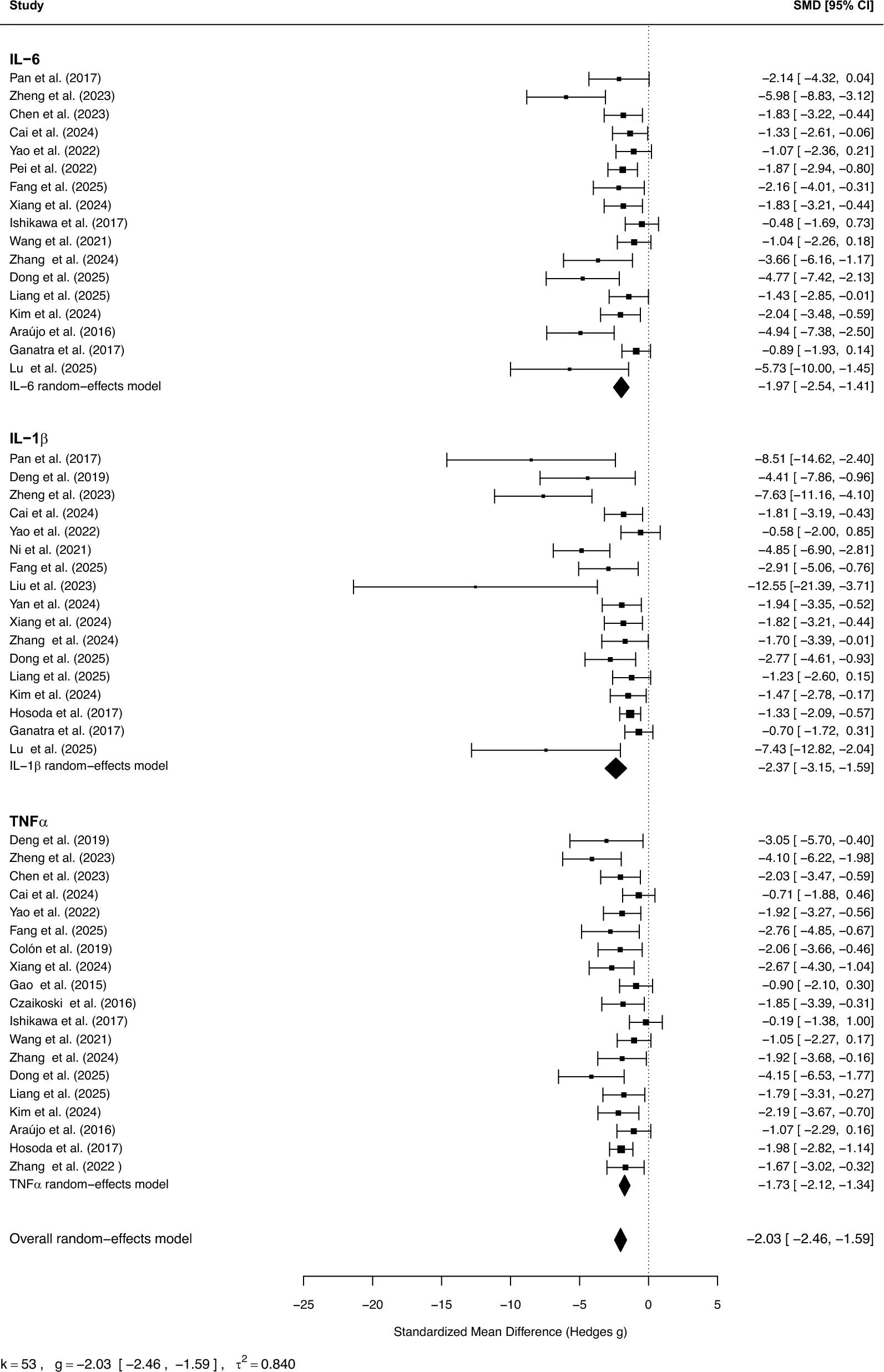
Forest plot of cytokine levels. The plot shows effect size with negative g values indicating a reduction in cytokine levels and 95% confidence interval limits. The black square represents the SEM (Hedge g) for each study, and its size is proportional to the study’s statistical weight. The black diamond presents the pooled Random-effect size for this model

Overall, the NETs modulation therapies showed a reduction in pro-inflammatory cytokine levels (Fig. 5). For IL-6, the pooled effects indicated a consistent, large decrease in the treated group compared to the control group. There was moderate heterogeneity, though statistically significant variability (k=17; g=-1.97; 95% CI:[-2.54,-1.41]; I^2^=53.6%; τ^2^=0.6926; Q(16)=35.83, p=0.003). For IL-1ꞵ levels, the model showed a large decrease in cytokine concentration in the treated population versus the control population. And with substantial and statistically significant heterogeneity in the analysis (k=17; g=-2.37; 95% CI:[-3.15,-1.59]; I^2^=70.6%; τ^2^=1.5686; Q(16)=47.46, p<0.001). Therapeutic intervention significantly reduces the pooled effect of TNF-ɑ levels when compared to the control, with only low, non‑significant heterogeneity (k=19; g=-1.73; 95% CI:[-2.13,-1.34]; I^2^=27.9%; τ^2^=0.2017; Q(18)=26.45, p=0.090).

### Tissue injury

**Fig. 6:**
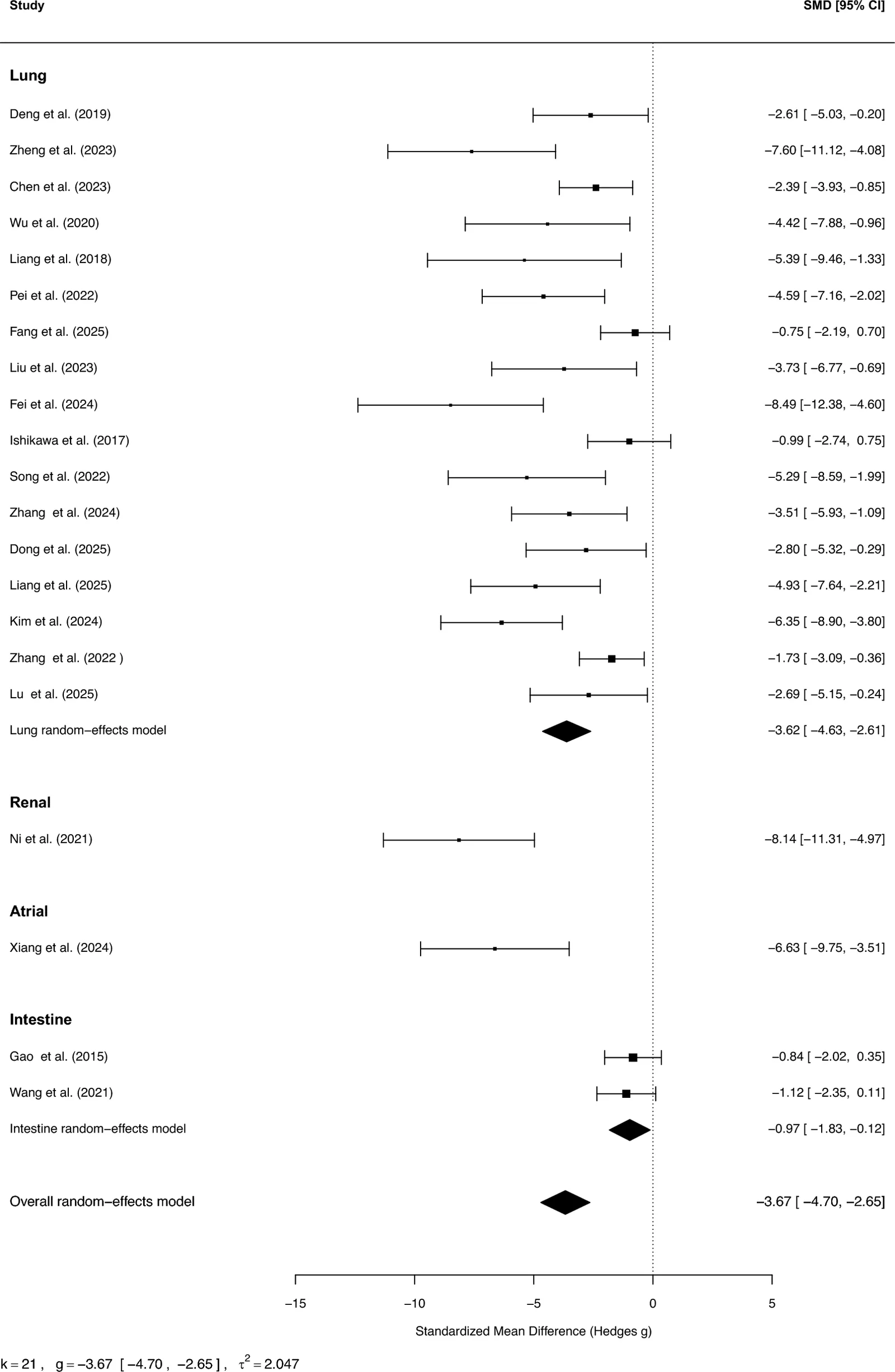
Forest plot of tissue injury via histology analysis. The plot shows effect size, with negative g values indicating reduced tissue injury, along with 95% confidence interval limits. The black square represents the SEM (Hedge g) for each study, and the size is proportional to the statistical weight of the study. The black diamond presents the pooled Random-effect size for this model

Sepsis causes widespread organ damage due to dysregulated immune response, which is visualized with histological staining of tissues. The meta-analysis revealed a significant overall decrease in tissue injury with NETs reduction therapy (Fig. 6; g=-3.67; 95% CI:[-4.70, -2.65]). However, there was substantial between‑study heterogeneity indicating considerable variability in effect sizes (I^2^=78.08%; τ^2^=4.0931; Q(20)=79.85, p<0.0001). To account for heterogeneity, tissue type was tested as a categorical moderator and significantly improved model fit (QM(3)=10.84, p=0.0126). These results indicate that tissue type is a moderator for effect size, but the limited number of studies, high heterogeneity, and subjective outcome assessment mean this effect should be interpreted with caution.

### Meta-regression of NETs reduction and outcomes

**Fig. 7:**
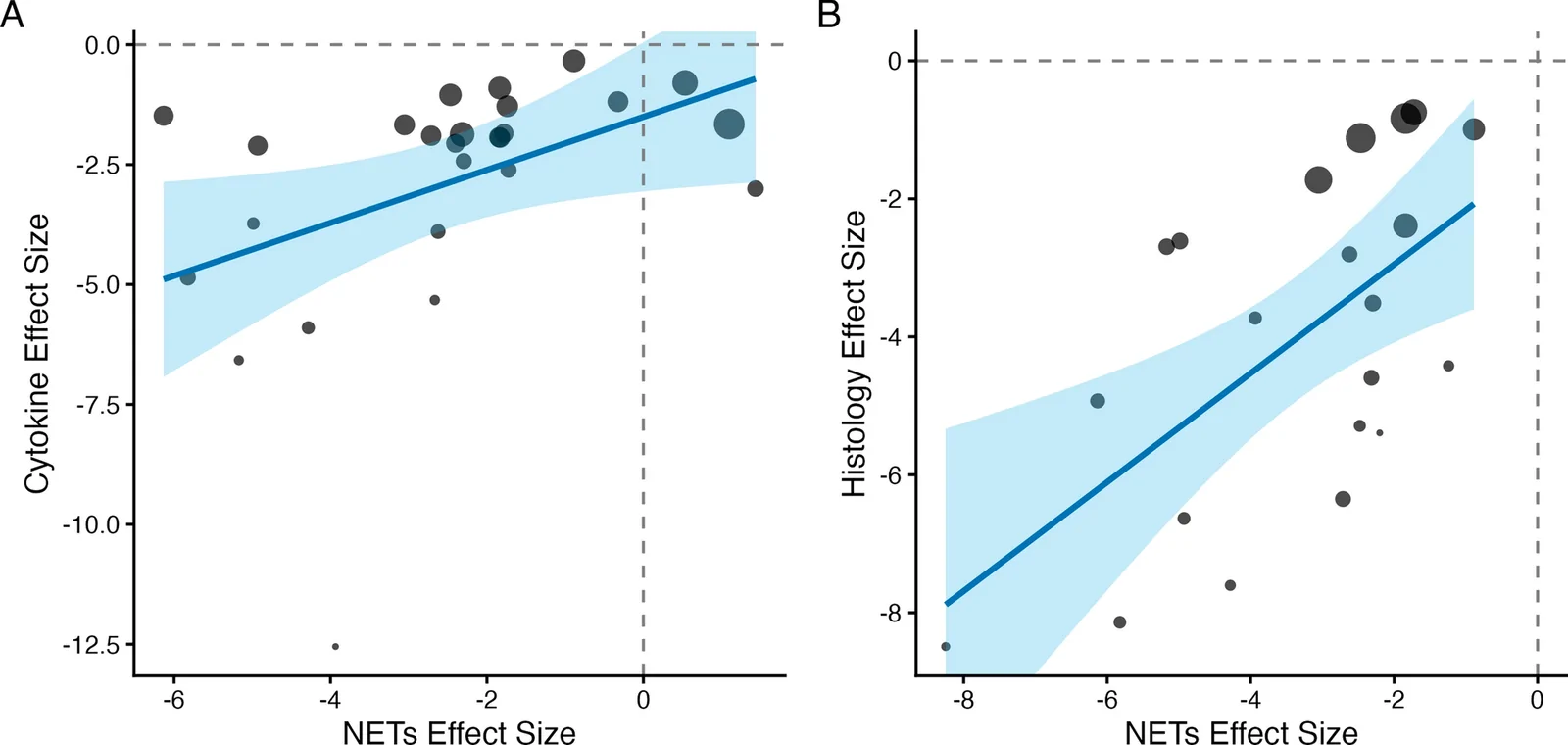
Association of NETs reduction with cytokine and histological outcomes. Scatter plots show relationships between NETs effect sizes and (**A**) cytokine and (**B**) histological effect sizes. Point size reflects inverse-variance weighting. Lines represent fitted meta-regression models with 95% confidence intervals

A meta-regression indicated that NETs levels reduction was significantly associated with improvements in outcomes. A greater reduction in NETs was associated with a larger decrease in cytokine levels (Fig. 7A; β=0.24; 95% CI:[0.05,0.43], p=0.013). The NETs effect size explains the 28% between-study heterogeneity, while significant residual heterogeneity remained (QE=43.1, p=0.01). Similarly, NETs reductions were strongly associated with improvements in histological outcomes (Fig. 7B; β=0.84, 95% CI:[0.36,1.31], p=0.0006). NETs reduction explained 50% of the between-study heterogeneity, although substantial residual heterogeneity remained (QE=47.5, p=0.0003).

### Publication bias

**Fig. 8:**
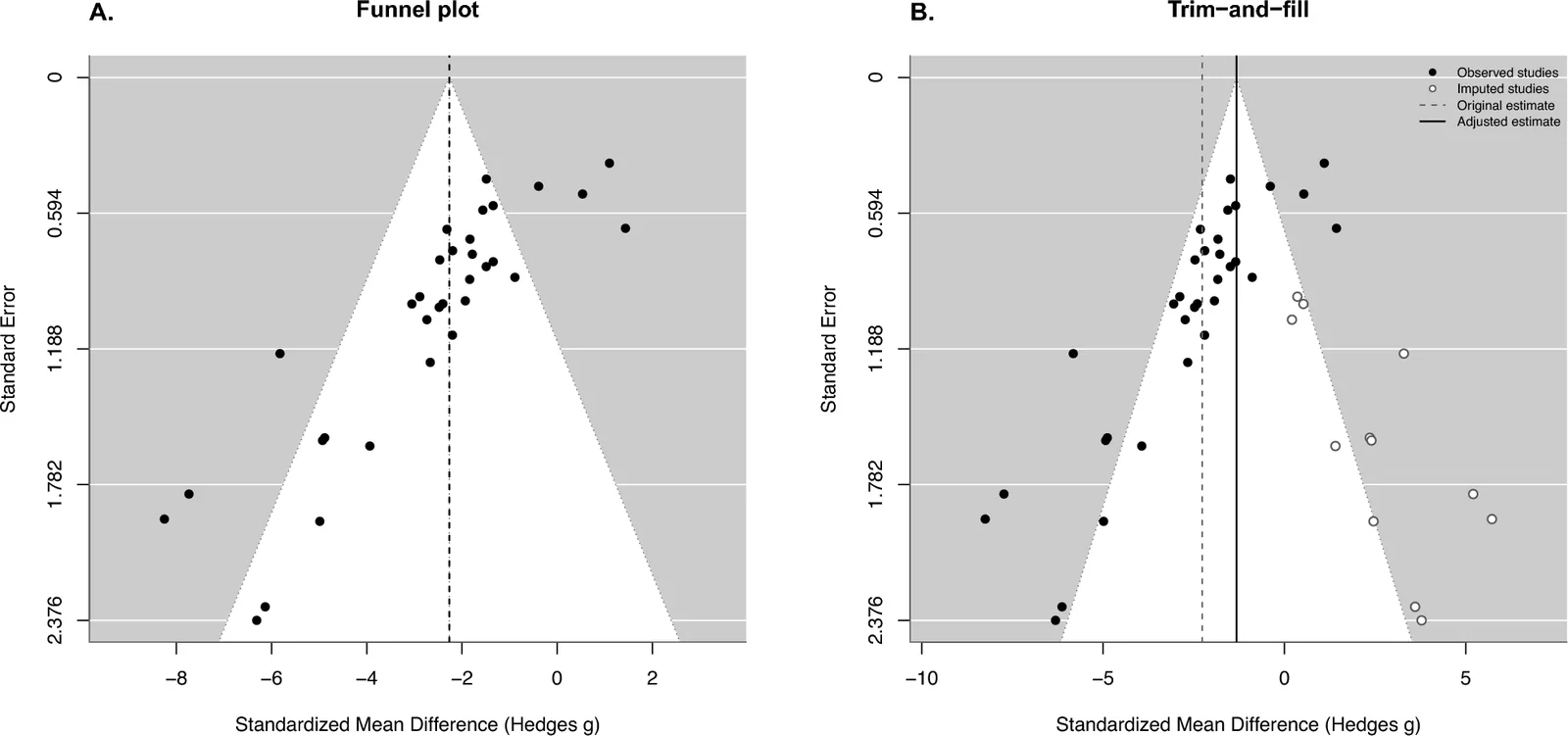
Funnel plots.** A**) Publication bias. **B**)Trim-and-fill analysis. Each filled point represents a study, and an open point represents an imputed study. The x-axis is the effect size (Hedge g), with negative values indicating a lower NETs level, thus favoring the therapy. The y-axis shows the standard error (SE). The vertical dotted line represents the pooled random effects estimate, and the diagonal lines correspond to the pseudo-95% confidence limits

We constructed a funnel plot using NETs levels to assess publication bias. Based on the NETs tier classification, we selected one NETs marker corresponding to the highest tier reported by the study (Table 1). This plot is based on NETs levels, as mortality outcomes were not consistently reported across studies (23 of 30). The funnel plot showed clear asymmetry, supported by Egger’s regression test, suggesting evidence of small study effects and publication bias (Fig. 8; k=33; Z=-7.61, p<0.00​01). Trim-and-fill analysis imputed 12 studies missing from the right side of the plot (Fig. 8). The pooled effect remained significant (g=-1.31; 95% CI:[-2.11,-0.53], p<0.001) compared to uncorrected estimate (g=-2.27; 95% CI:[-2.93,-1.60], p <0.001). Given the considerable heterogeneity observed (I^2^ =88%), the trim-and-fill results should be interpreted cautiously.

## Discussion

The main finding of this systematic review and meta-analysis was that modulation of NETs may improve outcomes in sepsis and LPS rodent models. NETs therapies are associated with reduced mortality and lower pro-inflammatory cytokine concentrations. Also, therapies attenuated tissue injury, although with substantial heterogeneity. Previous studies suggest that modulating NETs may be beneficial in treating sepsis, as dysregulated NETs may increase disease severity [11]. Coupled with the knowledge that sepsis accounts for millions of deaths worldwide, we found it paramount that science understands the intersection of NETs-targeting therapies and their impact on sepsis [2].

The included studies demonstrated that NETs therapies may improve animal outcomes. However, NETs exhibit a context-dependent role in inflammation. During infection, NETs formation may contribute to host defense by trapping and limiting the dissemination of pathogens. In contrast, in sterile inflammatory settings, excessive NETs formation can promote tissue injury and amplify inflammatory responses [19]. Because this review primarily relied on LPS-induced inflammatory models, caution is warranted when extrapolating these findings to other infectious or non-infectious inflammatory conditions. Future studies using diverse models of sepsis will be important to determine the broader applicability of our observations. Regardless, this detailed evaluation of underlying modulators (NETs tier, intervention timing, and therapeutic type) together with broader outcome measures, such as mortality, may provide insights into NETs and sepsis.

The animal models were relatively homogeneous, with studies predominantly consisting of mice and only two using rats. As a result, species differences were unlikely to contribute meaningfully to the observed heterogeneity. Despite our broad search strategy, most studies relied on intraperitoneal LPS administration to induce disease, which led to a subgroup analysis of non-LPS sepsis models. Another notable feature of the dataset was the variability in outcome timing. Most often, continuous mechanistic outcomes at early time points (<24 hours) were evaluated, whereas survival outcomes were assessed over extended periods (>96 hours). This review reflects early-phase NETosis biology resulting in a temporal disconnect between experimental and clinical endpoints.

The NETs tier quantification table provides a novel framework for future NETs analysis. However, NETs tiers did not emerge as a statistically significant moderator in NETs biomarker levels. Tiers 1 and 2, which used the most specific NETs markers (CitH3, MPO/NE-DNA complexes), produced comparable effect sizes (-1.77 and -2.17, respectively). In contrast, tier 3 yielded a substantially larger effect (-3.43), but the CIs indicated greater within-tier variability. The magnitude observed in tier 3 may be inflated because nonspecific markers overestimate the reduction, potentially misrepresenting the NETs reduction. These levels and biological consequences should be considered cautiously. Together, the variability of NETs quantification could be a limiting factor in the translation of NETs-targeting sepsis therapies.

A detailed analysis of intervention timing revealed that 45% of the studies used prophylactic treatments, reflecting a common but problematic approach in experimental research. Despite this, the sub‑analysis examining treatment timing as a moderator showed no statistically significant differences in NETs levels among treatments administered before, concurrent with, or after disease induction. Limiting the sensitivity analysis to the therapeutic interventions (48%) still demonstrated a significant reduction. However, the different intervention protocols highlight a distinct flaw when comparing prophylactic and therapeutic drugs due to mechanistic differences, as it may be the prevention of NETs formation and not the reduction of NETs improving outcomes. Ultimately, these models do not represent the same systematic effect or scenario; however, the results warrant further testing in models more aligned with clinical care.

NETosis is a multi-step process, and prophylactic treatments can hinder pathway analysis. However, we still categorized interventions that targeted NETs formation as inhibitors, those that targeted NETs clearance as degraders, and those that promoted NETs as agonists. Only a small number of included studies examined NETs degradation with DNase, an established approach for eliminating NETs, though it may also have side effects [20]. In contrast, approximately 70% of interventions were NETs inhibitors, predominantly targeting PAD4 activity and chromatin decondensation. Only three heterogeneous studies analyzed NETs agonists, but the pooled analysis continues to support that modulating NETs improves disease outcomes in animal models. Collectively, these findings indicate that current therapeutic strategies in the field are largely oriented toward preventing NETs formation rather than enhancing their clearance or increasing them.

Controlling for moderators did not change the overall pattern of NETs levels. All but three studies showed a consistent decrease in NETs concentrations, and together the pooled estimate effect favored treatment. However, the effect reflects the average across NETs-targeted interventions as a broad, heterogeneous category, which may obscure the mechanistic effects and physiological benefits of the therapies. Simultaneously, this review aimed to correlate NETs modulation with improvements in animal outcomes. However, because experimental studies often report outcomes inconsistently, we focused on the most frequently measured outcomes (mortality, pro-inflammatory cytokines, and tissue injury). Most of the included studies demonstrated that NETs reduction resulted in decreased mortality. This finding aligns with previous research stating that degrading NETs, inhibiting NETs, or blocking NETs pathogenic effects are promising methods to limit the impact of dysregulated NETs in sepsis [21]. Notably, three studies demonstrated reduced mortality despite increased NETs levels, which is interpreted as preliminary evidence that the effects of NETs depend on the biological context. While not all studies assessed cytokine levels, 17 reported reductions in IL-6 and IL-1β, and 19 reported reductions in TNF-α levels. This reduction in pro-inflammatory cytokines is important and compatible with our hypothesis, but there is a complex causality between cytokines and NETs levels that cannot be established with this meta-analysis. But previous research has shown that a feedback loop involving cytokines and NETs may lead to disease progression through dysregulated NETs stimulation [22]. In the included studies, the interventions reduced tissue injury, but the effect size was moderated by tissue type. Also, small study numbers and unblinded histological scoring mean findings should be considered exploratory. To explore the mechanistic relationship, meta-regression analyses indicated that treatment effects on NETs levels were significantly associated with decreasing cytokine concentrations and tissue injury. It is important to note that these outcomes are likely interrelated and may reflect shared underlying biological mechanisms rather than separate sources of evidence. Although these findings should be interpreted with caution, given the limitations of the studies, they demonstrate that greater reductions in NETs are associated with larger improvements in downstream outcomes. This finding encourages further research into NETs therapies.

This systematic review has several strengths. To our knowledge, this is the first review to provide a comprehensive overview of NETs modulation in experimental animal models, incorporating multiple intervention strategies to demonstrate the therapeutic potential. Also, this review followed PRISMA, and the protocol is published with INPLASY. This protocol used a binary key for study inclusion and a NETs tiered methodology, providing a framework and guidelines for future work. Moreover, stratified efficacy endpoint analysis of NETs, cytokines, mortality, and histology enabled a more complex understanding of the therapeutic influence on animal improvement.

This work also has limitations, consistent with the issues discussed in the executive summary of Minimum Quality Threshold in Preclinical Sepsis Studies (MQTiPSS) consensus [23]. Osuchowski et al. identify gaps and suggest recommendations to improve the standardization of experimental models. Using SYRCLE, we determined that most included studies were of poor methodological quality and not aligned with MQTiPSS. Even though the MQTiPSS and ARRIVE guidelines recommend clear and transparent study designs, many included studies lack protocols for rodent models, resulting in no universal intervention protocol or outcome measurements, insufficient reporting of randomization or inclusion of male and female animals, and publication bias [23, 24]. As evident from the publication bias, there are 12 potentially missing studies, and only three studies report findings of increased NETs levels. Thus, the results presented in this review must be interpreted cautiously, as the true effect may be smaller than estimated. Together, this highlights the need for more clinically relevant experimental protocols.

Several meta-analyses in this review reported statistical heterogeneity, suggesting that the studies differed substantially in models, protocols, or outcome assessment methods. MQTiPSS argues that challenging with LPS is not appropriate for human sepsis modeling and recommends using microorganisms commonly found in human sepsis [23]. Moreover, the MQTiPSS advises against prophylactic intervention protocols, as they do not replicate clinical care. Hence, this is a primary limitation for most of the studies and a motivation for improving modeling. Also, improved modelling may reduce limitations in dose-response analyses, as the LPS dose-response analysis was limited by insufficient meta-regression power, whereas therapeutic dose-response analysis was not feasible due to biological heterogeneity among interventions. As evidenced by our binary key and random effects modeling, most included studies reported a wide range of outcome measurements. Specifically, the studies reported the ordinal histological injury scores in bar-plots, forcing the extraction of the mean ± SD/SEM, making SMD the most appropriate effect size metric. Mortality was limited by the nature of the data provided, as HR were not possible, requiring subsequent meta-regression on follow-up duration and subgroup analysis. MQTiPSS’s suggestion to develop standardized criteria to monitor septic animals, along with a scoring system for the severity of organ/system dysfunction, would not only improve this meta-analysis but also greatly enhance the translation of experimental sepsis research into clinical outcomes.

## Conclusion

This systematic review and meta-analysis demonstrated that NETs modulation may be beneficial in experimental sepsis, with noted limitations. NETs-modulating therapies decrease mortality, pro-inflammatory cytokine levels, and tissue injury predominantly by reducing NETs; however, a small subset showed that increased NETs levels improve animal outcomes, suggesting therapeutic benefit may depend on optimal NETs regulation rather than suppression. While these significant findings are promising, their applicability is limited by the animal-study setting, methodological heterogeneity, and the bias identified in the review. Future research should focus on translating these therapies into more clinically relevant protocols. Ultimately, this review concluded that NETs modulation shows potential in sepsis and endotoxemia rodent models, while translation into clinical benefit will require more rigorous and relevant sepsis models.

## Supplementary Information

Additional file1

## Data Availability

Additional data and materials are available upon request
