# Supplementary material for: Treating sepsis by targeting neutrophil extracellular traps: a systematic review and meta-analysis
Source: Intensive Care Med Exp. 2026 Nov 4;14:144. doi: 10.1186/s40635-026-00994-2 (PMC13737313; doi:10.1186/s40635-026-00994-2)
Supplement: Supplementary file 1 [file 40635_2026_994_MOESM1_ESM.docx]

**Supplementary Figure 1. Search String**

PubMed search string:

( "neutrophil extracellular trap"[Title/Abstract]

OR "neutrophil extracellular traps"[Title/Abstract]

OR "NET formation"[Title/Abstract]

OR "NET release"[Title/Abstract]

OR NETosis[Title/Abstract]

OR NETotic[Title/Abstract] )

AND

(“ sepsis[Title/Abstract]

OR septic[Title/Abstract]

OR "septic shock"[Title/Abstract]

OR endotoxemia[Title/Abstract]

OR endotoxaemia[Title/Abstract]

OR bacteremia[Title/Abstract]

OR bacteraemia[Title/Abstract])

AND

("cecal ligation and puncture"[Title/Abstract]

OR "caecal ligation and puncture"[Title/Abstract]

OR CLP[Title/Abstract]

OR "cecal slurry"[Title/Abstract]

OR "caecal slurry"[Title/Abstract]

OR lipopolysaccharide*[Title/Abstract]

OR LPS[Title/Abstract]

OR endotoxin*[Title/Abstract]

OR "polymicrobial sepsis"[Title/Abstract]

OR "bacterial infection"[Title/Abstract])

AND ("2015/01/01"[Date - Publication] : "2025/05/31"[Date - Publication])

NOT review[Publication Type])

Scopus search string:

TITLE-ABS-KEY ( "neutrophil extracellular trap*" OR "NET formation" OR NETosis ) AND TITLE-ABS-KEY ( sepsis OR septic* OR endotox?emia OR bacter?emia ) AND TITLE-ABS-KEY ( CLP OR "cecal slurry" OR "caecal slurry" OR lipopolysaccharide* OR LPS OR endotoxin* OR "bacterial infection" ) AND PUBYEAR > 2014 AND PUBYEAR < 2025 AND NOT DOCTYPE ( re )

**Supplementary Figure 2. Binary Key for Study Inclusion**

|  | Type of therapy | | Net Quantification | | Cytokines | | IHC | | | | Survival | | |
| --- | --- | --- | --- | --- | --- | --- | --- | --- | --- | --- | --- | --- | --- |
| Study Number | A* | B | A | B | A | B | A | B | Tie | A | | B |  |
| 1 | 1 | 1 | 1 | 1 | 1 | 1 | 1 | 1 |  | 1 | | 1 |  |
| 2 | 1 | 1 | 1 | 1 | 1 | 1 | 1 | 1 |  | NA | | NA |  |
| 3 | 1 | 1 | 1 | 1 | 0 | 0 | 1 | 1 |  | 1 | | 1 |  |
| 4 | 1 | 1 | 1 | 1 | 1 | 1 | 1 | 1 |  | 1 | | 1 |  |
| 5 | 1 | 1 | 1 | 1 | 1 | 1 | 1 | 1 |  | NA | | NA |  |
| 6 | 1 | 1 | 1 | 1 | 1 | 1 | 1 | 1 |  | NA | | NA |  |
| 7 | 1 | 1 | 1 | 1 | 1 | 1 | NA | NA |  | NA | | NA |  |
| 8 | 1 | 1 | 1 | 1 | 1 | 1 | 1 | 1 |  | 1 | | 1 |  |
| 9 | 2 | 2 | 1 | 1 | NA | NA | NA | NA |  | 1 | | 1 |  |
| 10 | 1 | 1 | 1 | 1 | 1 | 1 | 1 | 1 |  | NA | | NA |  |
| 11 | 1 | 1 | 1 | 1 | 1 | 1 | 1 | 1 |  | NA | | NA |  |
| 12 | 1 | 1 | 1 | 1 | 1 | 1 | 1 | 1 |  | 1 | | 1 |  |
| 13 | 1 | 1 | 1 | 1 | 1 | 1 | 1 | 1 |  | 1 | | 1 |  |
| 14 | 1 | 1 | 1 | 1 | NA | NA | 1 | 1 |  | 1 | | 1 |  |
| 15 | 1 | 1 | 1 | 1 | 1 | 1 | 1 | 1 |  | 1 | | 1 |  |
| 16 | 1 | 1 | 1 | 1 | 1 | 1 | 1 | 1 |  | 1 | | 1 |  |
| 17 | 1 | 1 | 1 | 1 | NA | NA | 1 | 1 |  | NA | | NA |  |
| 18 | 1 | 1 | 1 | 1 | 1 | 1 | 1 | 1 |  | 1 | | 1 |  |
| 19 | 2 | 2 | 1 | 1 | 1 | 1 | 1 | 1 |  | 1 | | 1 |  |
| 20 | 1 | 1 | 1 | 1 | NA | NA | 1 | 1 |  | 1 | | 1 |  |
| 21 | 2 | 2 | 1 | 1 | 1 | 1 | 1 | 1 |  | 1 | | 1 |  |
| 22 | 2 | 2 | 1 | 1 | 1 | 1 | 1 | 1 |  | NA | | NA |  |
| 23 | 2 | 2 | 1 | 1 | 1 | 1 | 1 | 1 |  | NA | | NA |  |
| 24 | 2 | 2 | 1 | 1 | 1 | 1 | NA | NA |  | 1 | | 1 |  |
| 25 | 1 | 1 | 1 | 1 | NA | NA | NA | NA |  | 1 | | 1 |  |
| 26 | 2 | 2 | 1 | 1 | 1 | 1 | 1 | 1 |  | NA | | NA |  |
| 27 | 1 | 1 | 1 | 1 | 1 | 1 | 1 | 0 | 1 | 1 | | 1 |  |
| 28 | 1 | 1 | 1 | 1 | 1 | 1 | 1 | 1 |  | 1 | | 1 |  |
| 29 | 1 | 1 | 1 | 1 | 1 | 1 | 1 | 1 |  | 1 | | 1 |  |
| 30 | 1 | 1 | 1 | 1 | 1 | 1 | 1 | 1 |  | 1 | | 1 |  |
| 31 | 3 | 3 | 0 | 0 | 1 | 1 | NA | NA |  | 1 | | 1 |  |
| 32 | 3 | 3 | 0 | 0 | 1 | 1 | NA | NA |  | 1 | | 1 |  |
| 33 | 3 | 3 | 0 | 0 | 1 | 1 | NA | NA |  | 1 | | 1 |  |

*A and B are reviewers for study inclusion

**Supplementary Figure 3. GRADE Assessment**

| Outcome | No. of Studies (k) | Effect Estimate (95% CI) | Certainty Assessment | Overall Certainty of Evidence |
| --- | --- | --- | --- | --- |
| Mortality | 23 | RR 0.53 (0.46 to 0.62) | Risk of bias (-1) Indirectness (-1)  Publication bias (-1) Large effect (+1) | ⨁⨁⨁◯ MODERATE |
| NETs  Levels | 33 | SMD -2.27 (-2.93 to  -1.60) | Risk of bias (-1) Indirectness (-1) Inconsistency (-1) Publication bias (-1) Large effect (+1) | ⨁⨁◯◯ LOW |
| Tissue Injury | 21 | SMD -3.67 (-4.70 to  -2.65) | Risk of bias (-1) Indirectness (-1) Inconsistency (-1) Publication bias (-1) Large effect (+1) | ⨁⨁◯◯ LOW |
| IL-6 | 17 | SMD -1.97 (-2.54 to  -1.41) | Risk of bias (-1) Indirectness (-1)  Inconsistency (-1) Publication bias (-1) Large effect (+1) | ⨁⨁◯◯ LOW |
| TNF-α | 19 | SMD -1.73 (-2.12 to  -1.34) | Risk of bias (-1)  Indirectness (-1) Inconsistency (-1) Publication bias (-1) Large effect (+1) | ⨁⨁◯◯ LOW |
| IL-1β | 17 | SMD -2.37 (-3.15 to  -1.56) | Risk of bias (-1) Indirectness (-1) Inconsistency (-1) Publication bias (-1) Large effect (+1) | ⨁⨁◯◯ LOW |
| Key   a. Risk of bias: Most studies had an unclear or high risk of bias in at least one SYRCLE domain (random sequence generation, allocation concealment, blinding of investigators and outcome assessors, and sample size calculations) b. Indirectness: Findings were derived from experimental animal models of sepsis and may not fully capture the biological complexity and heterogeneity of human sepsis. c. Inconsistency: Secondary outcomes-substantial methodological and biological heterogeneity across studies (sepsis models, timing of administration, NET biomarkers, cytokine measurements, and histological scoring systems. Mortality- effect estimates were directionally consistent and low heterogeneity. d. Publication bias: Funnel plot asymmetry and small-study effects e. Large effect: Mortality and secondary outcomes- substantial effect sizes favoring NET-targeting interventions | | | | |
|  |  |  |  |  |
